# Some power allocation algorithms for cognitive uplink satellite systems

**DOI:** 10.1186/s13638-023-02234-7

**Published:** 2023-04-06

**Authors:** Arthur Louchart, Ehsan Tohidi, Philippe Ciblat, David Gesbert, Eva Lagunas, Charly Poulliat

**Affiliations:** 1 Centre for Digital Systems, IMT Nord Europe, Institut Mines-Télécom, University of Lille, Lille, France; 2 Department of Telecommunication Systems, Technical University of Berlin, Berlin, Germany; 3 Département Communications & Electronique, Télécom Paris, Institut Polytechnique de Paris, Palaiseau, France; 4 Communication Systems Department, EURECOM, Sophia-Antipolis, France; 5 Interdisciplinary Centre for Security, Reliability and Trust, University of Luxembourg, Luxembourg City, Luxembourg; 6 IRIT, Toulouse INP–ENSEEIHT, University of Toulouse, Toulouse, France

**Keywords:** Satellite systems, Cognitive radio, Distributed estimation, Signomial programming

## Abstract

Cognitive satellite communication (SatCom) is rapidly emerging as a promising technology to overcome the scarcity of the exclusive licensed band model in order to fulfill the increasing demand for high data rate services. The paper addresses power allocation methods for multi-operator multi-beam uplink satellite communication systems co-existing with a Ka-band terrestrial network, using cognitive radio paradigm. Such a scenario is especially challenging because of (i) the coexisting multiple SatCom operators over the cognitive band need to coordinate the use of their resources under limited inter-operator information exchange, and (ii) nonlinear onboard high power amplifier (HPA) which leads to nonlinear interference between users and beams. In order to tackle the first challenge, we propose distributed power allocation algorithms including the standard Alternate Direction Multiplier Method (ADMM); Regarding the HPA nonlinear impairment, we propose nonlinear-aware power allocation based on Signomial Programming. The proposed solutions outperform state-of-the-art in both cases.

## Introduction

The ongoing digital transformation has undoubtedly impacted the population’s expectations and demand for new interactive internet-based services. A lot of expectations are riding on the upcoming generation of wireless communications for such broadband applications. However, access to broadband technology in rural and remote parts of the Earth is still an unresolved issue. The economic impact and the social benefits that Internet brings shall be available anywhere and anytime in order to bridge the ever-wider digital divide. The latter has been shown to be accentuated with the COVID-19 pandemic, which revealed that lack of reliable and affordable internet access is not only affecting developing countries but also low-income communities around the world, including those in large urban areas.

Internet-by-satellite, also known as satellite broadband, represents a cost-efficient competitive solution for expanding ubiquitous broadband connectivity. During the 90 s, the satellite industry started launching the first High Throughput Satellites (HTS) into orbit, providing far more throughput than existing wideband satellites. Since then, the increase in demand for data rate has not stopped, resulting in a constant hunger for (i) more bandwidth, and (ii) better utilization of the bandwidth, i.e. spectral efficiency.

Traditionally, most of the existing systems operate on exclusive spectrum bands which are not shared with other entities. Due to spectrum scarcity and the dearth of high-impact techniques to enhance data rate, a promising approach is to extend the usable spectrum by considering operation in the non-exclusive bands. In that context, *World Radiocommunication Conference*, European Telecommunications Standards Institute (ETSI), and International Telecommunication Union-Radiocommunication Sector (ITU-R) have validated the co-primary use of certain spectrum portions in the Ka-band, i.e., 17.7$-$19.7 GHz (satellite downlink) and 27.5$-$29.5 GHz (satellite uplink). Particularly for the uplink, electronics communication commission (ECC) Decision (05) 01 has established the conditions under which 27.5$-$29.5 GHz spectrum can be used by cognitive Fixed Satellite Services (FSS) which, however, are not imposed and countries may choose to opt-out, leaving the spectrum regulation aspects uncertain depending on the geographical region. Since these frequencies are already occupied by incumbent terrestrial systems, called Fixed Services (FS) systems, the upcoming satellite-based systems will have to co-exist with them. The ITU proposes the segregation of the band in a recommendation [4], which is of course not spectrum efficient. A more intelligent approach is to use Cognitive Radio (CR) paradigm [5]. This paradigm may be implemented into three different approaches: (i) the overlay approach which, as a drawback, requires exchanges between satellite systems and existing FS systems; (ii) the interweave approach which, as a drawback, requires the FSS users to observe their environment to predict the appropriate transmission times; and (iii) the underlay approach which requires to know the average channels between FS and FSS transmitters. As suggested in [6], it is possible to meet the ITU regulation rules and so to guarantee the quality of service of existing FS systems, by deploying the underlay approach. Therefore we focus on this approach in this paper.

**Fig. 1:**
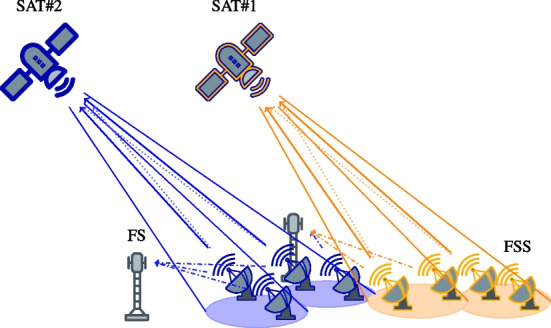
A multi-operator cognitive UL SatCom system composed of two operators. Each satellite has two beams, represented by ellipses, and two users (FSS) per beams. The dotted lines represent the inter-beam interference and the dashdotted lines represent the interference on primary users (FS) created by secondary users (FSS)

The cognitive users have thus to ensure that the impact of interference on the incumbent system does not exceed the regulatory interference limitations. In the particular instance of the SatCom CR system, the primary user is the incumbent terrestrial network (i.e., the FS) and the secondary users are the satellite terminals of interest, so-called FSS. As in more traditional terrestrial CR systems, one central issue to facilitate the co-existence between the CR devices and the incumbent is the *power allocation* in order to fulfill the cognitive radio constraints and to increase the data rate of the secondary systems. The SatCom CR systems described in Fig. 1 consider orthogonal access schemes within multiple beams, single color frequency reuse (i.e. all the beams use the same bandwidth), take into account HPA at the satellite side, and assume multiple satellite terminals belonging to different satellite operators (thus communicating to different Geostationary (GSO) satellites). Moreover, interference can affect multiple FS receivers. Notice that we are interested in the worst-case scenario of stressed satellite system, using the same frequency band for all beams, and allowing the high-power amplifier to be used in nonlinear regime.

Our SatCom CR system is thus closely related to allocation problems encountered in terrestrial-only CR systems. One can mention a strong link with the multi-cell Orthogonal Frequency Division Multiple Access (OFDMA) system or with Cloud Radio Access Network (CRAN), where a beam can be seen as a cell, the beam antenna as a base station, and the satellite as a BaseBand Unit (BBU). For instance, multi-cell OFDMA systems have been widely optimized in [7] (and references therein) but without the interference temperature constraints. In [8], the interference temperature constraints have been considered but the data rate does not undergo the multi-cell interference and the problem is straightforwardly convex over the power’s variables.

It is nevertheless different from terrestrial-only CR systems and so challenging for at least two reasons:

- The multiple operators where each satellite/BBU belongs to a different operator which does not have a ultra-high capacity backhaul between other operators and also does not wish to share sensitive information for privacy and security reasons. This underlines the need for *distributed* power allocation.
- The nonlinearity undergone by the signal retransmitted by the satellite due to nonlinear effect induced by some devices such as on-board High Power Amplifier (HPA). This causes *nonlinear* interference (on the secondary system). Even in absence of a cognitive radio scenario, managing power allocation in presence of nonlinear effect is a difficult issue. In fact, the developed method in this paper is general and can be applied in other contexts (i.e. without cognitive radio or without satellite transmitter), as long as a nonlinear effect can be modeled by a Volterra series. This is the core contribution of the paper.

Initial studies such as [6, 9, 10] have only partially covered the SatCom CR systems where single satellite operator and linear (or even zero) interference at the satellite side are considered. For instance, in [10], a heuristic worst-case approach has been proposed for power allocation. In [11], the authors have handled the sum-rate maximization by jointly optimizing the power allocation and beamforming, for the downlink of a satellite-terrestrial integrated network. Notice that the beamforming technique has been also considered for the downlink of some other cognitive satellite-based systems as in [12, 13]. Nevertheless, this additional technique is out of scope of this paper. Concerning the resource allocation taking into account the nonlinear interference, only a few works exist, not dealing with SatCom CR but rather OFDM based communications [14–18]. In addition, the way they have their specific optimization problem was swarm optimization [14], 1D search [15], and heuristic approach [16]. In [17], the authors have considered a nonlinear problem for only a class of nonlinear interference, which does not apply to our system model. In [19], the authors have focused on energy efficiency criterion while taking into account the nonlinear distortion, modeled using a third-order polynomial. In [18], it has been mentioned that the third-order nonlinear interference generated by the power amplifier could be managed through Geometric Programming (GP) for power optimization, but no simulations were performed. In this paper, we apply a general framework, the so-called Signomial Programming [20] for managing optimization problem with nonlinear effects.

The contributions of this paper are threefold: (i) improve performance compared to [6], (ii) consider distributed allocation in multiple operators setting, and (iii) take into account nonlinearity at the satellite side in the allocation algorithm. This last point is the main contribution of the paper.

Consequently, the paper is organized as follows: in Sect. 2, the system model is given and the general optimization problem is described. In Sect. 3, we consider the single operator case and the nonlinearity at the satellite side is neglected. This section corresponds to the first pillar of the paper in order to move then on more complicated cases. In Sect. 4 we move on the multiple operators setting where the centralized and distributed configurations are considered with a fair comparison in terms of data exchange between operators. In Sect. 5, we go back to single operator setting but by considering nonlinearities at the satellite side. Expressions of the data rate are given in closed-form and then the optimization problem is solved. In Sect. 6, numerical results are provided showing the relevance of the proposed algorithms. Concluding remarks and future works are drawn in Sect. 7.

## Methods–problem statement

As in DVB-RCS standard, we consider a Multiple-Frequency Time-Division Multiple Access (MF-TDMA). MF-TDMA allows a group of satellite terminals on the earth to communicate with the gateway (through the satellite of an operator) by means of a time-frequency resource grid. Essentially, a set of frequency carriers is considered, each of which is divided into time-slots. For the sake of synchronization aspects, fixed-slot MF-TDMA is usually considered, where the bandwidth and duration of successive traffic slots used by a particular terminal are fixed. In this paper, we work on the power allocation within one time-slot assuming that the subcarrier assignment has been already fixed. We consider a frequency reuse factor between each beam equal to one. In addition, we consider several satellites, each of them belonging to one operator. Each operator uses the same MF-TDMA scheme synchronized between them. If not synchronized, we may add a random time but the way to write the interference power between operators will be similar and does not modify the structure of the optimization problem, just its numerical evaluation. Therefore, for the sake of simplicity, we assume perfect synchronization.

### Signals closed-form expressions

We consider *P* operators. For each operator/satellite *p*, we consider *N* users spread over *B* beams, so there are $K:=N/B$ users using adjacent subbands in a beam. We assume that the subband assignment has been already done, and for sake of simplicity we abbreviate by user *k* the user using subband *k*. User *k* in beam *b* for satellite *p* will transmit a symbol sequence $\{a_{k,b,p,n}\}_{n}$. All users have the same shaping filter with an impulse response of $p_T(t)$. This shaping filter $p_T(t)$ is assumed to be a Square-Root Raised Cosine (SRRC) filter. We provide a list of symbols in Table 1.

**Table 1:** List of symbols

$\gamma _1$, $\gamma _3$	HPA nonlinear distortion coefficient
$\Delta F$	Frequency spacing
$T_s$	Symbol time
*B*	Number of beams
*K*	Number of secondary users per beam
*L*	Number of primary users (FS)
*N*	Number of secondary users (FSS)
*P*	Number of operators
*S*	Number of adjacent FSS subbands in one FS subband
*T*	Number of FS subbands
$H_{k}^{(j,p)}$	Channel response between user *k* of beam *j* for satellite *p* and antenna *j* of the same satellite
$H_{k}^{(b,j,p)}$	Channel response between user *k* of beam *b* for satellite *p* and antenna *j* of the same satellite
$H_{k}^{(b,q,j,p)}$	Channel response between user *k* of beam *b* for satellite *q* and antenna *j* of the satellite *p*
$I_{th}^{(\ell )}(m)$	Interference-temperature at FS $\ell$ on band interval *m*
$F_k^{(j,p,\ell )}$	Channel gain between user *k* belonging to beam *j* of satellite *p* to FS receiver $\ell$

Let $x_{k,b,p}(t)$ be the baseband signal emitted by the user *k* in beam *b* for satellite *p* whose expression is given by

1 $$\begin{aligned} x_{k,b,p}(t) = \sum _{n\in \mathbb{Z}} a_{k,b,p,n} p_T(t - n T_s). \end{aligned}$$

Each signal $x_{k,b,p}(t)$ is then transposed around the central frequency $f_k$ of the subband *k*. The difference between two adjacent frequencies is denoted by $\Delta F$.

The analytic signal on the antenna *j* associated with beam *j* of satellite *p*, denoted by $x_A^{(j,p)}(t)$, is the sum of the *K* analytic signals of this beam, the inter-beam interference denoted by $x_{A,IB}^{(j,p)}(t)$, and the inter-operator interference denoted by $x_{A,IP}^{(j,p)}(t)$.

2 $$\begin{aligned} x_A^{(j,p)}(t) =\sum _{k=1}^{K} H_{k}^{(j,p)} x_{k,j,p}(t) e^{i(2 \pi f_k t + \theta _k^{(j,p)})} + x_{A,IB}^{(j,p)}(t)+ x_{A,IP}^{(j,p)}(t) \end{aligned}$$

where

3 $$\begin{aligned} x_{A,IB}^{(j,p)}(t)=&\sum _{\begin{subarray}{c} b=1 \\ b\ne j \end{subarray}}^{B} \sum _{k=1}^{K} H_{k}^{(b,j,p)} x_{k,b,p}(t) e^{i(2 \pi f_k t + \theta _{k}^{(b,j,p)})}, \end{aligned}$$

4 $$\begin{aligned} x_{A,IP}^{(j,p)}(t)=&\sum _{\begin{subarray}{c} q=1 \\ q\ne p \end{subarray}}^{P} \sum _{b=1}^{B} \sum _{k=1}^{K} H_{k}^{(b,q,j,p)} x_{k,b,q}(t) e^{i(2 \pi f_k t+ \theta _{k}^{(b,q,j,p)})}, \end{aligned}$$

with

- $H_{k}^{(j,p)}$ and $\theta _{k}^{(j,p)}$, the complex-valued channel response and the carrier phase respectively between user *k* of beam *j* for satellite *p* and antenna *j* of the same satellite.
- $H_{k}^{(b,j,p)}$ and $\theta _{k}^{(b,j,p)}$, the complex-valued channel response and the carrier phase respectively between user *k* of beam *b* for satellite *p* and antenna *j* of the same satellite,
- $H_{k}^{(b,q,j,p)}$ and $\theta _{k}^{(b,q,j,p)}$, the complex-valued channel response and the carrier phase respectively between user *k* of beam *b* for satellite *q* and antenna *j* of the satellite *p*.

In this paper, we assume that the orbital positions of the satellites are far enough apart, enabling to neglect the inter-operator interference at the satellite side, since the beams for satellite *q* are directed on the satellite *q* and the energy spread on the direction of satellite *p* is incremental [21]. This leads to

5 $$\begin{aligned} H_k^{(b,q,j,p)}\approx 0, \quad \forall k,b,j,q\ne p, \end{aligned}$$

and thus

6 $$\begin{aligned} x_{A,IP}^{(j,p)}(t) \approx 0, \quad \forall j,p. \end{aligned}$$

Let $y_A^{(j,p)}(t)$ be the received analytic signal at the gateway coming from the antenna *j* of the satellite *p*. We assume one HPA per antenna for each satellite. And we also assume an Additive White Gaussian Noise (AWGN) channel between the satellite and the gateway. Consequently, according to [22, 23], we get

7 $$\begin{aligned} y_A^{(j,p)}(t) = \gamma _1 x_A^{(j,p)}(t) + \gamma _3 x_A^{(j,p)}(t) x_A^{(j,p)}(t) \overline{x_A^{(j,p)}}(t) + w_A(t), \end{aligned}$$

where $\overline{\; \cdot \; }$ stands for the complex-conjugate, and $w_A(t)$ is a complex-valued circularly-symmetric zero-mean additive white Gaussian noise with variance $\mathcal{P}_W$. The coefficients $\gamma _1$ and $\gamma _3$ are complex-valued parameters and characterize the nonlinear distortion of the HPA [23].

Let us now consider the demodulation for user *k* of beam *j* for satellite *p*. We first go back to baseband,

8 $$\begin{aligned} y_k^{(j,p)}(t) = y_A^{(j,p)}(t) e^{-i(2 \pi f_k t + \theta _{k}^{(j,p)}) }, \end{aligned}$$

we then apply the matched filter $p_R(t):=\overline{p_T}(-t)$,

9 $$\begin{aligned} z_k^{(j,p)}(t) = \int _{\mathbb{R}} p_R(\tau ) y_k^{(j,p)}(t - \tau ) d\tau . \end{aligned}$$

Finally, the signal is sampled at the symbol rate, resulting in the sequence $z_{k,n}^{(j,p)}$,

10 $$\begin{aligned} z_{k,n}^{(j,p)} = z_k^{(j,p)}(n T_s). \end{aligned}$$

By assuming perfect synchronization between beams, after a straightforward computation putting Eqs. (1–8) into Eq. (9), we have [3]

11 $$\begin{gathered} z_{k}^{{(j,p)}} (t) = \gamma _{1} \sum\limits_{{k^{\prime} = 1}}^{K} {\sum\limits_{{n^{\prime} \in \mathbb{Z}}} {H_{{k^{\prime}}}^{{(j,p)}} } } a_{{k^{\prime} ,j,p,n^{\prime}}} e^{{i(2\pi (f_{{k^{\prime}}} - f_{k} )t + \theta _{{k^{\prime} }}^{{(j,p)}} - \theta _{k}^{{(j,p)}} )}} h_{1} (t - n^{\prime}T_{s} ,k^{\prime} - k) \hfill \\ \quad + \gamma _{1} \sum _{{\begin{subarray}{*{20}c} {b = 1} \\ {b \ne j} \\ \end{subarray} }}^{B} \sum\limits_{{k^{\prime} = 1}}^{K} {\sum\limits_{{n^{\prime} \in \mathbb{Z}}} {H_{{k^{\prime}}}^{{(b,j,p)}} } } a_{{k^{\prime} ,b,p,n^{\prime}}} e^{{i(2\pi (f_{{k^{\prime} }} - f_{k} )t + \theta _{{k^{\prime} }}^{{(b,j,p)}} - \theta _{k}^{{(j,p)}} )}} h_{1} (t - n^{\prime}T_{s} ,k^{\prime} - k) \hfill \\ \quad + \gamma _{3} \sum\limits_{{b_{1} ,b_{2} ,b_{3} = 1}}^{B} {\sum\limits_{{k_{1} ,k_{2} ,k_{3} = 1}}^{K} {\sum\limits_{{n_{1} ,n_{2} ,n_{3} \in \mathbb{Z}}} {H_{{k_{1} }}^{{(b_{1} ,j,p)}} } } } H_{{k_{2} }}^{{(b_{2} ,j,p)}} \overline{{H_{{k_{3} }}^{{(b_{3} ,j,p)}} }} a_{{k_{1} ,b_{1} ,p,n_{1} }} a_{{k_{2} ,b_{2} ,p,n_{2} }} \overline{{a_{{k_{3} ,b_{3} ,p,n_{3} }} }} {\text{ }}\quad \times e^{{i(2\pi (k_{1} + k_{2} - k_{3} - k)\Delta Ft + \theta _{{k_{1} }}^{{(b_{1} ,j,p)}} + \theta _{{k_{2} }}^{{(b_{2} ,j,p)}} - \theta _{{k_{3} }}^{{(b_{3} ,j,p)}} - \theta _{k}^{{(j,p)}} )}} \hfill \\ \quad \times h_{3} (t - n_{1} T_{s} ,t - n_{2} T_{s} ,t - n_{3} T_{s} ,k_{1} + k_{2} - k_{3} - k)\quad + \int_{\mathbb{R}} {p_{R} } (\tau )w_{A} (t - \tau )e^{{ - 2i\pi f_{k} (t - \tau )}} d\tau , \hfill \\ \end{gathered}$$

with the following two Volterra kernels of first-order and third-order respectively,

12 $$\begin{aligned} h_1(t_1, \ell ) =&\int _{\mathbb{R}} p_T(t_1 - \tau ) p_R(\tau ) e^{-2 i \pi \ell \Delta F \tau } d \tau , \end{aligned}$$

13 $$\begin{aligned} h_3(t_1, t_2, t_3, \ell ) =&\int _{\mathbb{R}} p_T(t_1 - \tau ) p_T(t_2 - \tau ) p_T(t_3 - \tau ) p_R(\tau ) e^{-2 i \pi \ell \Delta F \tau } d \tau . \end{aligned}$$

Consequently, the term $z_{k,n}^{(j,p)}$ can be decomposed into four parts:

14 $$\begin{aligned} z_{k,n}^{(j,p)}=z_{k,n}^{(j,p),\text{L}}+ z_{k,n}^{(j,p),\text{I}}+ z_{k,n}^{(j,p),\text{NL}} + w_{k,n}^{(j,p)}, \end{aligned}$$

where $z_{k,n}^{(j,p),\text{L}}$ is the part depending on the current symbol, $z_{k,n}^{(j,p),\text{I}}$ is the part depending linearly on the symbols $\{a_{k,b,p,n}\}$ except the current one, and $z_{k,n}^{(j,p),\text{NL}}$ is the part depending nonlinearly on the symbols $\{a_{k,b,p,n}\}$.

As $h_1(nT_s,k)$ is zero for any $n\ne 0$ or any $k\ne 0$ (orthogonality in time and between users), and one otherwise, we force $k' = k$ and $n' = n$ to obtain the linear parts as follows

15 $$\begin{aligned}&z_{k,n}^{(j,p),\text{L}} = \gamma _1 H_{k}^{(j,p)} a_{k,j,p,n}, \end{aligned}$$

16 $$\begin{aligned}&z_{k,n}^{(j,p),\text{I}} = \gamma _1 \sum _{\begin{subarray}{c} b=1 \\ b \ne j \end{subarray}}^{B} H_{k}^{(b,j,p)} e^{i(\theta _{k}^{(b,j,p)} - \theta _{k}^{(j,p)})}a_{k,b,p,n}. \end{aligned}$$

The nonlinear part takes the following form

17 $$\begin{aligned}&z_{k,n}^{(j,p),\text{NL}} = \gamma _3 \sum _{b_1,b_2,b_3 = 1}^{B}\sum _{k_1,k_2,k_3=1}^{K} \sum _{n_1,n_2,n_3 \in \mathbb{Z}} H_{k_1}^{(b_1,j,p)} H_{k_2}^{(b_2,j,p)} \overline{H_{k_3}^{(b_3,j,p)}} \nonumber \\&\quad \times e^{ i(\theta _{k_1}^{(b_1,j,p)} + \theta _{k_2}^{(b_2,j,p)} - \theta _{k_3}^{(b_3,j,p)} - \theta _{k}^{(j,p)} )}\nonumber \\&\quad \times a_{k_1,b_1,p, n - n_1} a_{k_2,b_2,p,n - n_2} \overline{a_{k_3,b_3,p, n-n_3}} e^{2i \pi (k_1+k_2 - k_3-k) \Delta F n T_s} \nonumber \\&\quad \times h_3(n_1 T_s,n_2 T_s,n_3 T_s, k_1+k_2-k_3-k). \end{aligned}$$

In the rest of the paper, we will assume that the received signal of antenna *j* of satellite *p* at the gateway follows Eqs. (14–17). When only linearity is considered, we set $\gamma _1=1$ and $\gamma _3=0$ (see Sects. 3 and 4). Otherwise (see Sect. 5), $\gamma _1\ne 1$ and $\gamma _3\ne 0$.

### General optimization problem

We consider that the SatCom CR system adjusts its transmission strategy, i.e., its transmit power, with the goal of maximizing its own sum data rate while not causing harmful interference to the primary services [6, 24]. The reason behind this approach is that spectrum-hungry applications for SatCom are broadband services, which demand higher data rate.

It is well-known that maximizing data rate ignores fairness among different operators and users. Fairness objectives have been proposed in the literature to avoid such undesirable situations. Most of the works have considered fairness by focusing on the max-min or the sum-log of the cognitive user data rates [3, 10, 25, 26]. Here, the fairness between operators can be handle easily by focusing on a weighted sum data rate where the weights are chosen properly to compensate for the channels’ unfairness between operators if it exists. Fairness between users of the same operator is left for future works but adaptation to other figures of merit is feasible as done in [3, 26] for the single operator context.

Before focusing on the cost function to maximize, we first study the constraints. First of all, we need to limit the interference power received at each terrestrial incumbent (FS) receivers. We assume *L* primary FS receivers. As in [6], we assume that each primary receiver works on a set of band interval where each band interval corresponds to the set of *S* adjacent subbands of the SatCom CR system. We put $T=K/S$. For the sake of simplicity, we force *T* to be an integer. On each band interval $m\in \{1, \dots , T\}$ for each FS receiver $\ell \in \{1, \dots , L\}$, we have to satisfy the following interference-temperature constraints.

18 $$\begin{aligned} \sum _{p=1}^P \sum _{j=1}^B \sum _{k=(m-1)S+1}^{mS} F_k^{(j,p,\ell )} P_k^{(j,p)} \le I_{th}^{(\ell )}(m), \quad \forall \ell ,m, \end{aligned}$$

with

- $I_{th}^{(\ell )}(m)$ the interference-temperature at FS $\ell$ on band interval *m* that the SatCom CR system has to satisfy,
- $P_k^{(j,p)}:=\mathbb{E}[|a_{k,j,p,n}|^2]$ the power used by the user *k* belonging to beam *j* of satellite *p*,
- $F_k^{(j,p,\ell )}$ the channel gain between user *k* belonging to beam *j* of satellite *p* to FS receiver $\ell$.

In addition, for each user, we have a peak power constraint on each subband, i.e.,

19 $$\begin{aligned} 0\le P_k^{(j,p)}\le P_{\max }, \quad \forall k,j,p. \end{aligned}$$

We now move the general optimization problem corresponding to maximizing the weighted sum data rate of the whole SatCom CR systems satisfying the interference-temperature and individual power constraints. So we have Problem 1 where $R_k^{(j,p)}$ is the data rate for user *k* belonging to beam *j* of satellite *p*, and $\omega _p$ is the weight associated with operator *p* to ensure fairness between operators.

#### Problem 1

(Main Problem)

20 $$\begin{aligned}&\quad \left\{ P_k^{\star ,(j,p)} \right\} _{k,j,p} ={\mathop {\text{ arg max}}\limits _{\{P_k^{(j,p)}\}_{k,j,p}}}\, \sum _{p=1}^P \omega _p \left( \sum _{j=1}^B\sum _{k=1}^K R_k^{(j,p)} \right) \\ &\text {s.t. } (18) \text { and } (19) \nonumber . \end{aligned}$$

The way to express the data rate $R_k^{(j,p)}$ will depend on the sections since it depends on the receiver we carry out, i.e. the manner the linear and nonlinear interferences are treated.

Notice that the optimal solution is seldom full power $P_k^{(j,p)}=P_{\max }$ since the interference-temperature constraints as well as the linear and nonlinear interference (of the satellites on themselves) usually prevent this solution.

As we will see later, the involved functions in Problem 1 depend on the channels’ gains $G_k^{(b,j,p)}$ and $F_k^{(j,p,\ell )}$. The gains for the link between the satellite terminal and the satellite can easily be available since they depend on the user location which can be obtained through its position (using a Global Positioning System (GPS)) and the trajectory of the satellite which is known in advance. The gains for the links between the satellite terminals and the terrestrial devices can be listed into a database. Nevertheless, these values may be affected by strong fading in adverse weather conditions. If these events are short in time, they can be overcome using conventional higher-layer protocols like Automatic Request Retransmission (ARQ) [27].

## Basic problem: linear interference and single operator

In this Section, we consider the single operator setting, namely, $P=1$. Therefore, we omit the index *p* in the remainder of this Section. All the channels gains are assumed to be known.

Let us focus on the closed-form expression for $R_k^{(j)}$. We consider a separate inter-beam decoder where each beam is decoded by having only its own observations and by assuming the inter-beam interference as a noise. Consequently, the data rate writes as

21 $$\begin{aligned} R_k^{(j)}= \log _2\left( 1 + \frac{ G_k^{(j)} P_k^{(j)} }{\mathcal{P}_W +\sum \limits _{\begin{subarray}{c} b=1 \\ b\ne j \end{subarray}}^B G_{k}^{(b,j)} P_{k}^{(b)}}\right) \end{aligned}$$

where $P_k^{(j)}$ is the power assigned to user *k* belonging to beam *j* for the only satellite 1, $G_k^{(j)}=\left| H_k^{(j)}\right| ^2$ and $G_{k}^{(b,j)}=\left| H_{k}^{(b,j)}\right| ^2$ are the channel gains.

Consequently, the powers of the users sharing the same subband are coupled through the utility function (21) and the interference-temperature constraint (18). Optimizing (21) with constraint (18) is a nonconvex problem due to the utility function, but can be solved thanks to the well-known *Successive Convex Approximation* (SCA) method [28]. Actually, the inter-beam interference may be weak when the users sharing the same subband are far away from each other (and subband assignment not treated in this paper may force this property) or when the beams are well separate to each other (i.e., offer a negligible overlap). Therefore, the first idea comes from the possibility of neglecting the inter-beam interference in order to simplify the utility function [6, 10] in order to render this function convex. In that case, the figure of merit associated with the data rate of Eq. (21) is replaced with Eq. (22) stated in Problem 2.

### Problem 2

22 $$\begin{aligned} & \quad \left\{ P_k^{\star ,(j)} \right\} _{k,j} = {\mathop {\text{ arg max}}\limits _{ \{P_k^{(j)}\}_{k,j} }}\, \sum _{j=1}^B \sum _{k=1}^K \log _2\left( 1 + \frac{ G_k^{(j)}P_k^{(j)} }{\mathcal{P}_W}\right) , \\ &\text {s.t. } (18) \text { and } (19) \nonumber . \end{aligned}$$

The goal of this Section is now to solve Problem 2, which is convex. So the difficulty does not lie in the nature of the optimization problem but in the potential huge number of interference-temperature coupling constraints. To circumvent the use of standard toolboxes which converge slowly when the number of constraints is huge, some papers have proposed simplified algorithms [6, 10] by managing the coupling constraints in different ways. In [6], the interference-temperature constraints have been written beam-by-beam which decouples the optimization problem and enables the writing of a closed-form expression. This approach really makes sense when the number of FS receivers is small, but is less efficient if the FS receivers become dense as expected in the future. In [10], the authors have proposed to optimize the power by managing the worst case, i.e., the worst FS receiver receiving the maximum interference temperature when users are at full power, and then the power of the most interfering user is fixed for this FS receiver, by assuming that other users are full power, and so on.

Here, we propose a third way by taking into account the interference-temperature constraints one by one. After treating $\ell -1$ interference-temperature constraints, the solution is $P_k^{\star ,(j)}(\ell -1)$ (if $\ell =1$, we initialize $P_k^{\star ,(j)}(0)=P_{\max }$). Then the solution at iteration $\ell$ is obtained as the solution of Problem 3 given below.

### Problem 3

At iteration $\ell$, we have

23 $$\left\{ {P_{k}^{{{ \star },(j)}} (\ell )} \right\}_{{k,j}} = \mathop {\arg \max }\limits_{{\{ P_{k}^{{(j)}} \} _{{k,j}} }} \sum\limits_{{j = 1}}^{B} {\sum\limits_{{k = 1}}^{K} {\log _{2} } } \left( {1 + \frac{{G_{k}^{{(j)}} P_{k}^{{(j)}} }}{{{\mathcal{P}}_{W} }}} \right){\text{ }}$$

24 $$\begin{aligned}&\text {s.t. } \sum _{j=1}^B \sum _{k=(m-1)S+1}^{mS} F_k^{(j,\ell )} P_{k}^{(j)} \le I_{th}^{(\ell )}(m), \; \forall m, \end{aligned}$$

25 $$\begin{aligned}&0\le P_k^{(j)}\le P_k^{\star ,(j)}(\ell -1), \ \forall k,j. \end{aligned}$$

A waterfilling-like solution can be obtained for Problem 3. We get

26 $$\begin{aligned} P_k^{\star ,(j)}(\ell )= \left[ \frac{\mu _\ell (m_k)}{F_k^{(j,\ell )}} - \frac{\mathcal{P}_W}{G_k^{(j)}} \right] _{0}^{P_k^{\star ,(j)}(\ell -1)} \end{aligned}$$

where $[x]_{a}^b \!=\! \max (a,\min (b,x))$ for $a \!\le \! b$, $m_k:= \lceil k/S \rceil$ (with $\lceil \cdot \rceil$ the ceiling function) corresponds to the subband of the primary users disturbed by user *k*, and $\mu _\ell (m_k)$ is the water level chosen to fulfill the interference temperature constraint with equality. This approach is scalable into the number of FS since we have only *LT* waterfilling-like solutions to compute. The final solution is obtained as $P_k^{\prime ,(j)} = P_k^{\star ,(j)}(L)$. We will see in Sect. 6 that the proposed algorithm outperforms the existing ones and is close to the optimal solution in the context of SatCom CR systems.

## First main problem: multiple operators

In this Section, we consider the multiple operator setting, namely, $P>1$. We assume that each satellite operator is only aware of the channel information of its subscribed users. This information consists of the channel gain from the FSSs to the FSs (gain *F*) and from FSSs to the operator satellite (gain *G*), where the former affects the contribution on the interference level, while the latter has an impact on the utility function. Consequently, as long as the coupling constraints on interference level exist, information sharing is required to guarantee the QoS of the primary system. The main issue is: what level of information sharing may accept business-competing operators? This level raises both problems: the privacy (operator does not wish to share lot of information about its own subscribers. For instance, channel state information can be correlated back to the user location), and the complexity of the algorithm (the amount and so the time devoted to information exchange is limited).

Although earlier discussed challenge on power allocation (for instance, huge number of interference temperature constraints) still exists, in this section, we only focus on the coordination schemes (equivalently, the type and amount of information sharing) among the satellite operators and investigate the challenge on power allocation from this perspective. The optimization problem to be solved (if channels’ gains are known by a central network manager computing the solution) is as follows.

### Problem 4

27 $$\begin{aligned} \{P_k^{\star ,(j,p)}\}_{k,j,p}=&{\mathop {\text{ arg max}}\limits _{\{P_k^{(j,p)}\}_{k,j,p} }}\, \sum _{p=1}^P \omega _p \left( \sum _{j=1}^B\sum _{k=1}^K \log _2\left( 1+\frac{ G_k^{(j,p)} P_k^{(j,p)} }{\mathcal{P}_W +\sum \limits _{\begin{subarray}{c} b=1 \\ b\ne j \end{subarray}}^B G_{k}^{(b,j,p)} P_{k}^{(b,p)}}\right) \right) \end{aligned}$$

$$\text {s.t. } \, (19), $$

28 $$\begin{aligned}&\sum _{p=1}^P \sum _{j=1}^B \sum _{k=(m-1)S+1}^{mS} F_k^{(j,p,\ell )} P_{k}^{(j,p)} \le I_{th}^{(\ell )}(m), \quad \forall \ell ,m \end{aligned}$$

We remind that inter-operator interference at each satellite is neglected since each satellite has very directed communication pattern. Unlike Sect. 3, we keep for sake of generality the inter-beam interference of each operator since the main issue on which we will focus is the level of information sharing instead of the nature of the utility function.

Let us now consider three cases of information sharing level leading to three kinds of algorithms.

### No information sharing (NIS)

In the context of no information sharing, we need to decouple Problem 4 into subproblems for which each operator can optimize itself. As only the interference temperature constraints couple the operators, the idea is to split these constraints operator by operator. As the primary users can be located everywhere and not concentrated on specific beams of specific operators, we suggest to split the interference temperature in an equal manner for each operator. Consequently, each operator has to solve the following problem.

#### Problem 5

For operator/satellite *p*, we have

29 $$\begin{aligned} \{P_k^{\star ,NIS,(j,p)}\}_{k,j}=&{\mathop {\text{ arg max}}\limits _{\{P_k^{(j,p)}\}_{k,j} }}\, \sum _{j=1}^B\sum _{k=1}^K \log _2\left( 1+\frac{ G_k^{(j,p)}P_k^{(j,p)} }{\mathcal{P}_W +\sum \limits _{\begin{subarray}{c} b=1 \\ b\ne j \end{subarray}}^B G_k^{(b,j,p)} P_k^{(b,p)}}\right) \end{aligned}$$

$$\text {s.t. } \, (19), $$

30 $$\begin{aligned}&\sum _{j=1}^B \sum _{k=(m-1)S+1}^{mS}F_k^{(j,p,\ell )} P_{k}^{(j,p)} \le J_{th}^{(\ell )}(m), \quad \forall \ell ,m, \end{aligned}$$

$$\text {with } \, J_{th}^{(\ell )}(m)=\frac{I_{th}^{(\ell )}(m)}{P}.$$

One can remark that Problem 5 is similar to Problem 2 where the inter-beam interference has been kept. Problem 5 is a nonconvex optimization problem due to the denominator in the logarithm function. We solve it by using SCA approach. Again, we may remove the inter-beam interference term and then Problem 5 boils down to Problem 2 and the strategy used in Sect. 3 consisting in treating the interference temperature constraint one by one may be employed.

This method obviously vanishes the information sharing between operators but we may loose performance especially because each operator is treated independently and not jointly. This approach is fully distributed since each operator works independently.

### Channel information sharing (CIS)

In that case, we assume a centralized approach where a central node (typically a network manager) collects information about all the channel states.

We assume that the central node

- Has only *q*-bit quantified version of the channel states, namely, $G_k^{(j,p),q}$, $G_k^{(b,j,p),q}$, and $F_k^{(j,p,\ell ),q}$.
- Solve Problem 4 based on the quantified version of the channels’ gains. The solution is denoted by $P_{k}^{\star , CIS, (j,p)}$.
- Compute the thresholds for each operator *p* as
  31 $$\begin{aligned} J_{th}^{(p,\ell )}(m):= \sum _{j=1}^B \sum _{k=(m-1)S+1}^{mS} F_k^{(j,p,\ell ),q}P_{k}^{\star , CIS, (j,p)}. \end{aligned}$$
- Send a *q*-bit quantified version of $J_{th}^{(p,\ell )}(m)$ at satellite *p*, denoted by $J_{th}^{(p,\ell ),q}(m)$.

Then each operator knows its level of admissible interference temperature through $J_{th}^{(p,\ell ),q}(m)$. Each operator *p* thus solves Problem 5 with its true channels’ information ($G_k^{(j,p)}$, $G_k^{(b,j,p)}$, and $F_k^{(j,p,\ell )}$) and the threshold $J_{th}^{(p,\ell ),q}(m)$.

The total number of exchanged bits among operators and the central node for the proposed channel information sharing algorithm is

32 $$\begin{aligned} n_\text{exchanged} = qP(K B^2+LKB+ LT). \end{aligned}$$

since we count the upload of the channels gains and the download of the thresholds.

### Interference level information sharing

In this Section, we focus only on approaches sharing interference level instead of channel gains since the main bottleneck in our optimization problem is the coupling due to the interference temperature. Once the inference level is shared, each operator will solve its own optimization problem locally. This approach enables us to reduce the level of information exchange as well as to protect more the privacy as operators do not share the channels’ gains and do not unveil location information of their subscribers to the other operators. We consider two algorithms: *i)* the so-called Iterative Interference Level Sharing, and *ii)* one based on ADMM.

#### Iterative Interference Level Sharing (IILS)

In Problem 5, all the thresholds are split equally among the operators, however, a threshold can be a bottleneck for one operator (it means its interference temperature constraint is satisfied) while it is not limiting for another (the interference temperature constraint is not reached since power constraint is more restrictive). Thus, the idea is to share the global threshold on the operators differently. To do that, we calculate the power allocation for each operator with the equal splitting approach (actually the NIS algorithm). If the global threshold is not reached with this power allocation, the gap is shared equilikely between operators which leads to provide more opportunity to the operators satisfying the interference temperature constraint in the case of NIS algorithm. Consequently, new interference temperature thresholds are re-calculated, and then we iterate.

Consequently, at each iteration, we perform the NIS algorithm for the remaining amount of the allowed interference if not used totally the threshold.

We denoted by $J^{(p,\ell )}(m)_{|\textbf{P}^{(p)}}$ the interference imposed by the users of operator *p* on PU $\ell$ on subband *m* when the power allocation is $\textbf{P}^{(p)}:=\{P_k^{(j,p)}\}_{k,j}$, i.e.,

33 $$\begin{aligned} J^{(p,\ell )}(m)_{|\textbf{P}^{(p)}}=\sum _{j=1}^B \sum _{k=(m-1)S+1}^{m S} F_k^{(j,p,\ell )} P_{k}^{(j,p)}. \end{aligned}$$

At iteration *i*, we calculate the contribution of operator *p* on FS $\ell$ on subband *m* as Eq. (33) (which may be less than $J_{th}^{(p,\ell )}(m)$), but we have

34 $$\begin{aligned} \sum _{p=1}^P J^{(p,\ell )}(m)_{|\textbf{P}^{(p)}(i)} \leqslant I_{th}^{(\ell )}(m). \end{aligned}$$

Thus, the remaining space on subband *m* of FS $\ell$ is

35 $$\begin{aligned} I^{(\ell )}_\text{rem} (m)= I_{th}^{(\ell )}(m) - \sum _{p=1}^P J^{(p,\ell )}(m)_{|\textbf{P}^{(p)}(i)}. \end{aligned}$$

Consequently, the threshold of operator *p* for the next iteration will be

36 $$\begin{aligned} J_{th}^{(p,\ell )} (m) = \frac{I^{(\ell )}_\text{rem} (m)}{P} + J^{(p,\ell )}(m)_{|\textbf{P}^{(p)}(i)}. \end{aligned}$$

We repeat this procedure for a given number of iterations denoted by $n_\text{iter}$. We remark that the threshold for each operator increases if the global interference temperature budget was not completely used at the previous iteration.

This algorithm requires a total number of exchanged bits equal to

37 $$\begin{aligned} \begin{aligned} n_\text{exchanged}&= 2 n_\text{iter}qPLT, \end{aligned} \end{aligned}$$

where *q* is the number of bits for quantifying each threshold.

#### ADMM

This is a well-known iterative optimization algorithm that is well suited to distributed nonconvex optimization [29]. Here, we need to adapt the formulation of Problem 4 in order to force the coupling constraints to be in an equality form. By introducing new positive real-valued variables $$\varvec{\Delta } = \left( \Delta ^{(\ell ,m)}\right) _{\begin{subarray}{c} 1 \le \ell \le L \\ 1 \le m \le T \end{subarray}}$$ representing the remaining interference for primary user $\ell$ on subband *m*, we have

##### Problem 6

38 $$\begin{aligned}{} & {} \left\{ \{P_k^{\star ,(j,p)}\}_{k,j,p}, \mathbf {\Delta }\right\} = {\mathop {\text{arg max}}\limits _{\{\{P_k^{(j,p)}\}_{k,j,p}, \mathbf {\Delta }\}}}\, \sum _{p=1}^P \omega _p f_p(\{P_k^{(j,p)}\}_{k,j}) \end{aligned}$$

39 $$\begin{aligned}{} & {} \text {with } f_p(\{P_k^{(j,p)}\}_{k,j}) = \sum _{j=1}^B\sum _{k=1}^K \log _2\left( 1+\frac{ G_k^{(j,p)}P_k^{(j,p)} }{\mathcal{P}_W +\sum \limits _{\begin{subarray}{c} b=1 \\ b\ne j \end{subarray}}^B G_{k}^{(b,j,p)} P_{k}^{(b,p)}}\right) \end{aligned}$$

$$\text {s.t. } \, (19), $$

40 $$\begin{aligned}{} & {} \Delta ^{(\ell ,m)} + \sum _{p=1}^P I_p^{(\ell ,m)} = I_{th}^{(\ell )}(m), \quad \forall \ell ,m, \end{aligned}$$

41 $$\begin{aligned}{} & {} 0\le \Delta ^{(\ell ,m)} \le I_{th}^{(\ell )}(m), \quad \forall \ell , m, \end{aligned}$$

where $I_p^{(\ell ,m)} = \sum _{j=1}^B \sum _{k=(m-1)S+1}^{m S} F_k^{(j,p,\ell )} P_{k}^{(j,p)}$ is the interference on primary user $\ell$ in subband *m*, created by operator *p*. The resulting augmented Lagrangian is given by

42 $$\begin{aligned} L_{\rho } \left( \{P_k^{(j,p)}\}_{k,j,p}, \varvec{\Delta }, \varvec{\lambda } \right) =&- \sum _{p=1}^P \omega _p \left( \sum _{j=1}^B\sum _{k=1}^K \log _2\left( 1+\frac{ G_k^{(j,p)}P_k^{(j,p)} }{\mathcal{P}_W +\sum \limits _{\begin{subarray}{c} b=1 \\ b\ne j \end{subarray}}^B G_{k}^{(b,j,p)} P_{k}^{(b,p)}}\right) \right) \nonumber \\&+ \sum _{\ell =1}^{L} \sum _{m=1}^{M} \lambda ^{(\ell ,m)} \left( \Delta ^{(\ell ,m)} + \sum _{p=1}^P I_p^{(\ell ,m)} - I_{th}^{(\ell )}(m) \right) \nonumber \\&+ \frac{\rho }{2} \sum _{\ell =1}^{L} \sum _{m=1}^{M} \left( \Delta ^{(\ell ,m)} + \sum _{p=1}^P I_p^{(\ell ,m)} - I_{th}^{(\ell )}(m) \right) ^2 \end{aligned}$$

where $\rho \!>\! 0$ is a constant penalty parameter and $$\varvec{\lambda }=\left( \lambda ^{(\ell ,m)} \right) _{\begin{subarray}{c} 1 \le \ell \le L \\ 1 \le m \le T \end{subarray}}$$ are the Lagrangian multipliers.

Therefore, following the algorithm presented in [29], at iteration *i*, the algorithm consists of there steps:

- The first (local) step in which operator *p* computes the following optimization problem
  43 $$\begin{aligned}{} & {} \{P_{k}^{\star ,ADMM,(j,p)}(i)\}_{k,j} = {\mathop {\text{arg min}}\limits _{\{P_k^{(j,p)}\}_{k,j}}}\, g_p(\{P_k^{(j,p)}\}_{k,j}) \end{aligned}$$
  44 $$\begin{aligned}{} & {} \text {with } g_p(\{P_k^{(j,p)}\}_{k,j}) = - \omega _p \sum _{j=1}^B\sum _{k=1}^K \log _2\left( 1+\frac{ G_k^{(j,p)}P_k^{(j,p)} }{\mathcal{P}_W +\sum \limits _{\begin{subarray}{c} b=1 \\ b\ne j \end{subarray}}^B G_{k}^{(b,j,p)} P_{k}^{(b,p)}}\right) \nonumber \\{} & {} \quad + \sum _{\ell =1}^{L} \sum _{m=1}^{M} \lambda ^{(\ell ,m)}(i-1) I_p^{(\ell ,m)} \nonumber \\{} & {} \quad + \frac{\rho }{2} \sum _{\ell =1}^{L} \sum _{m=1}^{M} \left( I_p^{(\ell ,m)} - I_p^{(\ell ,m)}(i-1) + \epsilon ^{(\ell ,m)}(i-1) \right) ^2, \end{aligned}$$
  $\text {s.t. } \, (19),$ where $\epsilon ^{(\ell ,m)}(i-1)$ and $\lambda ^{(\ell ,m)}(i-1)$ are the variables coming from central node at iteration $(i-1)$. Then each local node sends its interference contribution at iteration *i*, $I^{(\ell ,m)}_p(i)$, to the central node.
- The second (central) step in which the central node solves the following optimization problem
  45 $$\begin{aligned}{} & {} \varvec{\Delta }(i) = {\mathop {\text{arg min}}\limits _{\varvec{\Delta }}}\, h(\varvec{\Delta }) \end{aligned}$$
  46 $$\begin{aligned}{} & {} \text {with } h(\varvec{\Delta }) = \sum _{\ell =1}^{L} \sum _{m=1}^{M} \lambda ^{(\ell ,m)}(i-1) \Delta ^{(\ell ,m)} \nonumber \\{} & {} \quad + \frac{\rho }{2} \sum _{\ell =1}^{L} \sum _{m=1}^{M} \left( \Delta ^{(\ell ,m)} + \sum _{p=1}^P I_p^{(\ell ,m)}(i) - I_{th}^{(\ell )}(m) \right) ^2 \end{aligned}$$
  $$\text {s.t. } \, (41). $$
  This step just corresponds to solve second-order polynomials within an interval.
- The third (central) step in which the central node updates its local variables:
  47 $$\begin{aligned}&\epsilon ^{(\ell ,m)}(i) = \Delta ^{(\ell ,m)}(i) + \sum _{p=1}^P I_p^{(\ell ,m)}(i) - I_{th}^{(\ell )}(m), \quad \forall \ell , m, \end{aligned}$$
  48 $$\begin{aligned}&\lambda ^{(\ell ,m)}(i) = \lambda ^{(\ell ,m)}(i-1) + \rho \epsilon ^{(\ell ,m)}(i), \quad \forall \ell , m. \end{aligned}$$

Then the central node broadcasts its variables $$\varvec{\epsilon }(i) = \left( \epsilon ^{(\ell ,m)}(i) \right) _{\begin{subarray}{c} 1 \le \ell \le L \\ 1 \le m \le T \end{subarray}}$$ and $\varvec{\lambda }(i)$ to all local nodes.

Remind that all information exchange between the central and local nodes are quantified. This algorithm requires a total number of exchanged bits equal to

49 $$\begin{aligned} \begin{aligned} n_\text{exchanged}&= n_\text{iter}q (PLT + 2LT ), \end{aligned} \end{aligned}$$

where *q* is the number of bits for any quantified variable, and $n_\text{iter}$ is the number of iterations for the ADMM. The complexity of this algorithm is not studied here, however it is reduced to the resolution of a finite sequence of convex problems. The study of the complexity and convergence of convexity problems is done in [30, 31].

## Second main problem: nonlinear interference

In this Section, we would like to analyze and optimize the power allocation when nonlinear HPA occurs on board. For the sake of clarity, we go back to the single operator case ($P=1$). Nevertheless the extension to the multiple operators case can be done easily once again by neglecting the linear and nonlinear inter-operator interference (we remind that the linear inter-operator has been neglected in Sect. 4 which justifies to neglect also the nonlinear inter-operator interference.). As in Sects. 3–4, we consider the inter-beam (linear) interference but also an intra-beam (nonlinear) interference occur due to the nonlinear HPA.

According to Eq. (14), we remind that

50 $$\begin{aligned} z_{k,n}^{(j,p)}=z_{k,n}^{(j,p),\text{L}}+ z_{k,n}^{(j,p),\text{I}}+ z_{k,n}^{(j,p),\text{NL}} + w_{k,n}^{(j,p)}, \end{aligned}$$

where $z_{k,n}^{(j,p),\text{L}}$, $z_{k,n}^{(j,p),\text{I}}$, and $z_{k,n}^{(j,p),\text{NL}}$ are given by Eqs. (15), (16), and (17) respectively.

Assuming Gaussian-distributed symbols and gaussiannity for $z_{k,n}^{(j,p),\text{I}}$, and $z_{k,n}^{(j,p),\text{NL}}$ by the Central Limit Theorem, we have [32, 33] that the capacity for the user *k* on beam *j* associated with operator *p* (called data rate in the remainder of the paper due to the non-optimal assumption on the symbols distribution),

51 $$\begin{aligned} R^{(j)}_k \!=\! \log _2 \left( 1 + \frac{ \mathcal{P}_{\text {L},k}^{(j)^2} + 2 \mathcal{P}_{\text {L},k}^{(j)} \Re \{ \mathcal{P}_{\text {LNL},k}^{(j)} \} + |\mathcal{P}_{\text {LNL},k}^{(j)}|^2 }{ \mathcal{P}_{\text {L},k}^{(j)} \mathcal{P}_{\text {NL},k}^{(j)} + 2 \mathcal{P}_{\text {L},k}^{(j)} \Re \{ \mathcal{P}_{\text {INL},k}^{(j)}\} + \mathcal{P}_{\text {L},k}^{(j)} \mathcal{P}_{\text {I},k}^{(j)} + \mathcal{P}_{\text {L},k}^{(j)} \mathcal{P}_\text {W} - |\mathcal{P}_{\text {LNL},k}^{(j)}|^2 } \right) \end{aligned}$$

where we remove the superscript *p* related to the operator numbering (since only one operator is considered here), and where $\mathcal{P}_{\text {L},k}^{(j)}=\mathbb{E}[|z_{k,n}^{(j,p),\text{L}}|^2]$, $\mathcal{P}_{\text {NL},k}^{(j)}=\mathbb{E}[|z_{k,n}^{(j,p),\text{NL}}|^2]$, $\mathcal{P}_{\text {LNL},k}^{(j)}=\mathbb{E}[ z_{k,n}^{(j,p),\text{L}} \overline{z_{k,n}^{(j,p),\text{NL}}}]$, $\mathcal{P}_{\text {I},k}^{(j)}=\mathbb{E}[|z_{k,n}^{(j,p),\text{I}}|^2]$, $\mathcal{P}_{\text {INL},k}^{(j)}=\mathbb{E}[ z_{k,n}^{(j,p),\text{I}} \overline{z_{k,n}^{(j,p),\text{NL}}}]$, and $\mathcal{P}_\text {W}=\mathbb{E}[ |w_{k,n}^{(k,j)}|^2]$.

The first contribution of this Section is to find closed-form expressions for all the terms involved in Eq. (51). Before going further, we put some remarks:

- We first show that the denominator is positive thanks to Cauchy-Schwarz inequality for complex random variables since $|\mathcal{P}_{\text {LNL},k}^{(j)}|^2 \le \mathcal{P}_{\text {L},k}^{(j)} \mathcal{P}_{\text {NL},k}^{(j)}$.
- When $\gamma _3 = 0$, all the terms involving nonlinear parts vanish and the data rate becomes
  52 $$\begin{aligned} R_{\text {lin. interf.},k}^{(j)} = \log _2 \left( 1 + \frac{\mathcal{P}_{\text {L},k}^{(j)}}{ \mathcal{P}_\text {W} + \mathcal{P}_{\text {I},k}^{(j)}} \right) \end{aligned}$$
  and is obviously the same as in Eq. (21).
- If we assume that the receiver sees the nonlinear interference as an additional noise, i.e., the receiver is nonlinear-agnostic, then the data rate is given by
  53 $$\begin{aligned} \underline{R}_k^{(j)} = \log _2 \left( 1 + \frac{\mathcal{P}_{\text {L},k}^{(j)}}{ \mathcal{P}_\text {W} + \mathcal{P}_{\text {NL},k}^{(j)} + \mathcal{P}_{\text {I},k}^{(j)} } \right) \end{aligned}$$
  which is equivalent to put $\mathcal{P}_{\text {LNL},k}^{(j)} = 0$ and $\mathcal{P}_{\text {INL},k}^{(j)} = 0$ in Eq. (51).

### Closed-form expressions for the involved terms in data rate

The closed-form expressions of all the terms involved in the data rate expressions are the following ones.

54 $$\begin{aligned} \mathcal{P}_{\text{L},k}^{(j)}&= \left| \gamma _1\right| ^2 G_{k}^{(j)} P_{k}^{(j)} \end{aligned}$$

55 $$\begin{aligned} \mathcal{P}_{\text{I},k}^{(j)}&= \left| \gamma _1\right| ^2 \sum _{\begin{subarray}{c} b=1 \\ b \ne j \end{subarray}}^{B} G_{k}^{(b,j)} P_{k}^{(b)} \end{aligned}$$

56 $$\begin{aligned} \mathcal{P}_{\text{LNL},k}^{(j)}&= 2 \gamma _1 \overline{\gamma _3} \beta G_{k}^{(j)} P_{k}^{(j)} \sum _{k'=1}^{K} \sum _{b=1}^{B} G_{k'}^{(b,j)} P_{k'}^{(b)}, \end{aligned}$$

57 $$\begin{aligned} \mathcal{P}_{\text{INL},k}^{(j)}&= 2 \gamma _1 \overline{\gamma _3} \beta \sum _{k'=1}^{K} \sum _{\begin{subarray}{c} b=1 \\ b \ne j \end{subarray}}^{B} \sum _{b'=1}^{B} G_{k}^{(b,j)} G_{k'}^{(b',j)} P_{k}^{(b)} P_{k'}^{(b')}. \end{aligned}$$

where $$\beta = \sum _{n \in \mathbb{Z}} \overline{h_3}(0,nT_s,nT_s, 0)$$, and

58 $$\begin{aligned}&\mathcal{P}_{\text{NL},k}^{(j)} = 4 \left| \gamma _3\right| ^2 \alpha ^{(1)}_{0} \sum _{k',k''=1}^{K} \sum _{b_1,b_2,b_3=1}^{B} G_{k}^{(b_1,j)} G_{k'}^{(b_2,j)} G_{k''}^{(b_3,j)} P_{k}^{(b_1)} P_{k'}^{(b_2)} P_{k''}^{(b_3)} \nonumber \\&\quad + 4 \tilde{\delta }_{k,K} \left| \gamma _3\right| ^2 \alpha ^{(1)}_{1} \sum _{k',k''=1}^{K} \sum _{b_1,b_2,b_3=1}^{B} G_{k+1}^{(b_1,j)} G_{k'}^{(b_2,j)} G_{k''}^{(b_3,j)} P_{k+1}^{(b_1)} P_{k'}^{(b_2)} P_{k''}^{(b_3)} \nonumber \\&\quad + 4 \tilde{\delta }_{k,1} \left| \gamma _3\right| ^2 \alpha ^{(1)}_{1} \sum _{k',k''=1}^{K} \sum _{b_1,b_2,b_3=1}^{B} G_{k-1}^{(b_1,j)} G_{k'}^{(b_2,j)} G_{k''}^{(b_3,j)} P_{k-1}^{(b_1)} P_{k'}^{(b_2)} P_{k''}^{(b_3)} \nonumber \\&\quad + 2 \left| \gamma _3\right| ^2 \alpha ^{(2)}_{0} \sum _{\begin{subarray}{c} k_1,k_2,k_3 =1 \\ k=k_1+k_2-k_3 \end{subarray}}^{K} \sum _{b_1,b_2,b_3=1}^{B} G_{k_1}^{(b_1,j)} G_{k_2}^{(b_2,j)} G_{k_3}^{(b_3,j)} P_{k_1}^{(b_1)} P_{k_2}^{(b_2)} P_{k_3}^{(b_3)} \nonumber \\&\quad + 2 \left| \gamma _3\right| ^2 \alpha ^{(2)}_{1} \sum _{\begin{subarray}{c} k_1,k_2,k_3 =1 \\ k=k_1+k_2-k_3 \pm 1 \end{subarray}}^{K} \sum _{b_1,b_2,b_3=1}^{B} G_{k_1}^{(b_1,j)} G_{k_2}^{(b_2,j)} G_{k_3}^{(b_3,j)} P_{k_1,}^{(b_1)} P_{k_2}^{(b_2)} P_{k_3}^{(b_3)}, \end{aligned}$$

with $$\tilde{\delta }_{k,k'} =1-\delta _{k,k'}$$, $$\alpha ^{(1)}_{\ell } = \sum _{n_1 \in \mathbb{Z}} \left| \sum _{n_2 \in \mathbb{Z}} h_3(n_1 T_s,n_2 T_s,n_2 T_s, \ell ) \right| ^2$$ and $$\alpha ^{(2)}_{\ell } = \sum _{n_1,n_2,n_3 \in \mathbb{Z}} \left| h_3(n_1 T_s,n_2 T_s,n_3 T_s, \ell )\right| ^2$$.

Due to the space limitation, we will hereafter just provide the main steps for deriving $\mathcal{P}_{\text{NL},k}^{(j)}$. Other terms can be done in a similar way.

By using Eq. (17), we obtain that

59 $$\begin{aligned}&\mathcal{P}_{\text{NL},k}^{(j)} = \left| \gamma _3\right| ^2 \sum _{b_1,b_2,b_3 = 1}^{B} \sum _{b_1',b_2',b_3' = 1}^{B} \sum _{k_1,k_2,k_3=1}^{K} \sum _{k_1^{\prime},k_2^{\prime},k_3^{\prime}=1}^{K} \sum _{n_1,n_2,n_3 \in \mathbb{Z}} \sum _{n_1^{\prime},n_2^{\prime},n_3^{\prime} \in \mathbb{Z}} A \nonumber \\&\quad \times H_{k_1}^{(b_1,j)} H_{k_2}^{(b_2,j)} \overline{H_{k_3}^{(b_3,j)} H_{k_1^{\prime}}^{(b_1^{\prime},j)} H_{k_2^{\prime}}^{(b_2^{\prime},j)}} H_{k_3^{\prime}}^{(b_3^{\prime},j)} \nonumber \\&\quad \times e^{ i(\theta _{k_1}^{(b_1,j,p)} + \theta _{k_2}^{(b_2,j,p)} - \theta _{k_3}^{(b_3,j,p)} - \theta _{k}^{(j,p)} )} e^{ -i(\theta _{k_1^{\prime}}^{(b_1^{\prime},j,p)} + \theta _{k_2^{\prime}}^{(b_2^{\prime},j,p)} - \theta _{k_3^{\prime}}^{(b_3^{\prime},j,p)} - \theta _{k}^{(j,p)} )}\nonumber \\&\quad \times e^{2i \pi (k_1+ k_2 - k_3 - k) \Delta F n T_s} e^{-2 i\pi (k_1^{\prime} + k_2^{\prime} - k_3^{\prime} - k)\Delta F n T_s} \nonumber \\&\quad \times h_3(n_1 T_s,n_2 T_s,n_3 T_s, k_1+k_2-k_3-k) \overline{h_3}(n_1^{\prime} T_s,n_2^{\prime} T_s,n_3^{\prime} T_s, k_1^{\prime} + k_2^{\prime} - k_3^{\prime} - k) \end{aligned}$$

with $A= \mathbb{E}[ a_{k_1,b_1, n - n_1} a_{k_2,b_2,n - n_2} \overline{a_{k_3,b_3, n-n_3}} \overline{a_{k_1',b_1', n - n_1'}} \overline{a_{k_2',b_2',n - n_2'}} a_{k_3',b_3', n-n_3'}]$. As the symbols $\{a_{k,b,n}\}$ are assumed to be circularly-symmetric complex-valued Gaussian random variable, according to Isserlis’ theorem [34, 35], we have

60 $$\begin{aligned} A=A_1+A_2+A_3+A_4+A_4+A_5+A_6 \end{aligned}$$

with

$$\begin{aligned} \begin{gathered} A_1 =\mathbb{E} \left[ a_{k_1,b_1,n-n_1} \overline{a_{k_1',b_1',n-n_1'}} \right] \mathbb{E} \left[ a_{k_2,b_2,n-n_2} \overline{a_{k_3,b_3,n-n_3}} \right] \mathbb{E} \left[ \overline{a_{k_2',b_2',n-n_2'}} a_{k_3',b_3',n-n_3'} \right] , \\ A_2= \mathbb{E} \left[ a_{k_1,b_1,n-n_1} \overline{a_{k_1',b_1',n-n_1'}} \right] \mathbb{E} \left[ a_{k_2,b_2,n-n_2} \overline{a_{k_2',b_2',n-n_2'}} \right] \mathbb{E} \left[ \overline{a_{k_3,b_3,n-n_3}} a_{k_3',b_3',n-n_3'} \right] , \\ A_3= \mathbb{E} \left[ a_{k_1,b_1,n-n_1} \overline{a_{k_3,b_3,n-n_3}} \right] \mathbb{E} \left[ a_{k_2,b_2,n-n_2} \overline{a_{k_2',b_2',n-n_2'}} \right] \mathbb{E} \left[ \overline{a_{k_1',b_1',n-n_1'}} a_{k_3',b_3',n-n_3'} \right] , \\ A_4= \mathbb{E} \left[ a_{k_1,b_1,n-n_1} \overline{a_{k_3,b_3,n-n_3}} \right] \mathbb{E} \left[ a_{k_2,b_2,n-n_2} \overline{a_{k_1',b_1',n-n_1'}} \right] \mathbb{E} \left[ \overline{a_{k_2',b_2',n-n_2'}} a_{k_3',b_3',n-n_3'} \right] , \\ A_5= \mathbb{E} \left[ a_{k_1,b_1,n-n_1} \overline{a_{k_2',b_2',n-n_2'}} \right] \mathbb{E} \left[ a_{k_2,b_2,n-n_2} \overline{a_{k_3,b_3,n-n_3}} \right] \mathbb{E} \left[ \overline{a_{k_1',b_1',n-n_1'}} a_{k_3',b_3',n-n_3'} \right] , \\ A_6= \mathbb{E} \left[ a_{k_1,b_1,n-n_1} \overline{a_{k_2',b_2',n-n_2'}} \right] \mathbb{E} \left[ a_{k_2,b_2,n-n_2} \overline{a_{k_1',b_1',n-n_1'}} \right] \mathbb{E} \left[ \overline{a_{k_3,b_3,n-n_3}} a_{k_3',b_3',n-n_3'} \right] . \end{gathered} \end{aligned}$$

Consequently, we can split Eq. (59) into six terms as follows

61 $$\begin{aligned} \mathcal{P}_{\text{NL},k}^{(j)} = p_{\text{NL},k}^{(j)}(1)+ p_{\text{NL},k}^{(j)}(2)+p_{\text{NL},k}^{(j)}(3)+p_{\text{NL},k}^{(j)}(4)+p_{\text{NL},k}^{(j)}(5)+p_{\text{NL},k}^{(j)}(6) \end{aligned}$$

with, for $$i\in \{1,\cdots , 6\}$$,

62 $$\begin{aligned}&p_{\text{NL},k}^{(j)}(i)= \left| \gamma _3\right| ^2 \sum _{b_1,b_2,b_3 = 1}^{B} \sum _{b_1^{\prime},b_2^{\prime},b_3^{\prime} = 1}^{B} \sum _{k_1,k_2,k_3=1}^{K} \sum _{k_1^{\prime},k_2^{\prime},k_3^{\prime}=1}^{K} \sum _{n_1,n_2,n_3 \in \mathbb{Z}} \sum _{n_1^{\prime},n_2^{\prime},n_3^{\prime} \in \mathbb{Z}} A_i \nonumber \\&\quad \times H_{k_1}^{(b_1,b)} H_{k_2}^{(b_2,b)} \overline{H_{k_3}^{(b_3,b)} H_{k_1^{\prime}}^{(b_1^{\prime},b)} H_{k_2^{\prime}}^{(b_2^{\prime},b)}} H_{k_3^{\prime}}^{(b_3^{\prime},b)} \nonumber \\&\quad \times e^{ i(\theta _{k_1}^{(b_1,j,p)} + \theta _{k_2}^{(b_2,j,p)} - \theta _{k_3}^{(b_3,j,p)} - \theta _{k}^{(j,p)} )} e^{ -i(\theta _{k_1^{\prime}}^{(b_1^{\prime},j,p)} + \theta _{k_2^{\prime}}^{(b_2^{\prime},j,p)} - \theta _{k_3^{\prime}}^{(b_3^{\prime},j,p)} - \theta _{k}^{(j,p)} )}\nonumber \\&\quad \times e^{2i \pi (k_1+ k_2 - k_3 - k) \Delta F n T_s} e^{-2 i\pi (k_1^{\prime} + k_2^{\prime} - k_3^{\prime} - k)\Delta F n T_s} \nonumber \\&\quad \times h_3(n_1 T_s,n_2 T_s,n_3 T_s, k_1+k_2-k_3-k) \overline{h_3}(n_1^{\prime} T_s,n_2^{\prime} T_s,n_3^{\prime} T_s, k_1^{\prime} + k_2^{\prime}- k_3^{\prime} - k). \end{aligned}$$

One can easily show that $p_{\text{NL},k}^{(j)}(1) = p_{\text{NL},k}^{(j)}(5)$, $p_{\text{NL},k}^{(j)}(2) = p_{\text{NL},k}^{(j)}(6)$ and $p_{\text{NL},k}^{(j)}(3) = p_{\text{NL},k}^{(j)}(4)$ and implies that

63 $$\begin{aligned} \mathcal{P}_{\text{NL},k}^{(j)}= 2 p_{\text{NL},k}^{(j)}(1)+ 2 p_{\text{NL},k}^{(j)}(2)+ 2p_{\text{NL},k}^{(j)}(3). \end{aligned}$$

Once again, due to the space limitation, we focus hereafter only on the derivations of $p_{\text{NL},k}^{(j)}(1)$. Other terms $p_{\text{NL},k}^{(j)}(2)$ and $p_{\text{NL},k}^{(j)}(3)$ can be computed in a similar manner.

In each symbol expectation, the term is non-null only if both first indexes are equal to each other and if both second indexes are equal to each others. Consequently, $A_1$ is different from zero if we have $k_1=k_1^\prime$, $k_2=k_3$, $k_2'=k_3'$ and $b_1=b_1'$, $b_2=b_3$, $b_2'=b_3'$ and $n_1=n_1'$, $n_2=n_3$, $n_2'=n_3'$. So

64 $$\begin{aligned}{} & {} p_{\text{NL},k}^{(j)}(1) = \left| \gamma _3\right| ^2 \sum _{k_1,k_2,k_2'=1}^{K} \sum _{b_1,b_2,b_2'=1}^{B} \sum _{n_1,n_2,n_2' \in \mathbb{Z}} G_{k_1}^{(b_1,j)} G_{k_2}^{(b_2,j)} G_{k_2'}^{(b_2',j)} \nonumber \\{} & {} \quad \times P_{k_1}^{(b_1)} P_{k_2}^{(b_2)} P_{k_2'}^{(b_2')} h_3(n_1 T_s,n_2 T_s,n_2 T_s, k_1-k) \overline{h_3}(n_1 T_s,n_2' T_s,n_2' T_s, k_1 - k). \end{aligned}$$

According to [23], we keep only the dominant terms in $h_3$ by forcing the last entry to be zero, 1 or $-1$ (majority of the interference comes from the subband itself and its adjacent neighbors). This leads to the following decomposition of $p_{\text{NL},k}^{(j)}(1)$ into three terms

65 $$\begin{aligned} p_{\text{NL},k}^{(j)}(1) = p_{\text{NL},k,0}^{(j)}(1) + p_{\text{NL},k, 1}^{(j)}(1) + p_{\text{NL},k, -1}^{(j)}(1), \end{aligned}$$

with, for $$\varepsilon \in \{-1,0,1\}$$,

66 $$\begin{aligned}{} & {} p_{\text{NL},k,\varepsilon }^{(j)}(1) = \left| \gamma _3\right| ^2 \sum _{k_1,k_2,k_2'=1}^{K} \sum _{b_1,b_2,b_2'=1}^{B} \sum _{n_1,n_2,n_2' \in \mathbb{Z}} G_{k_1}^{(b_1,b)} G_{k_2}^{(b_2,b)} G_{k_2'}^{(b_2',b)} \nonumber \\{} & {} \quad \times P_{k_1,b_1} P_{k_2,b_2} P_{k_2',b_2'} \nonumber \\{} & {} \quad \times h_3(n_1 T_s,n_2 T_s,n_2 T_s, k_1-k = \varepsilon ) \overline{h_3}(n_1 T_s,n_2' T_s,n_2' T_s, k_1 - k = \varepsilon ). \end{aligned}$$

We then get

- Case $\varepsilon =0$ (intra-subband interference): As a consequence, we have
  67 $$\begin{aligned}{} & {} p_{\text{NL,0}}^{(1)}(k,b) = \left| \gamma _3\right| ^2 \sum _{b_1}^{B} G_{k_1}^{(b_1,b)} P_{k,b_1} \nonumber \\{} & {} \quad \times \sum _{b_2,b_2'=1}^{B} \sum _{k_2,k_2'=1}^{K} G_{k_2}^{(b_2,b)} G_{k_2'}^{(b_2',b)} P_{k_2,b_2} P_{k_2',b_2'} \nonumber \\{} & {} \quad \times \sum _{n_1 \in \mathbb{Z}} \sum _{n_2 \in \mathbb{Z}} \sum _{n_2' \in \mathbb{Z}} h_3(n_1 T_s,n_2 T_s,n_2 T_s, 0) \overline{h_3}(n_1 T_s,n_2' T_s,n_2' T_s,0). \end{aligned}$$
  By changing some indexes notations, we finally obtain
  68 $$\begin{aligned} p_{\text{NL},k,0}^{(j)}(1) = \left| \gamma _3\right| ^2 \alpha ^{(1)}_{0} \sum _{k',k''=1}^{K} \sum _{b_1,b_2,b_3=1}^{B} G_{k}^{(b_1,j)} G_{k'}^{(b_2,j)} G_{k''}^{(b_3,j)} P_{k}^{(b_1)} P_{k'}^{(b_2)} P_{k''}^{(b_3)}. \end{aligned}$$
- Case $\varepsilon =1$ (right adjacent subband interference): As a consequence, we have for $k \ne K$
  69 $$\begin{aligned} p_{\text{NL},k, 1 }^{(j)} (1) = \left| \gamma _3\right| ^2 \alpha ^{(1)}_{1} \sum _{k',k''=1}^{K} \sum _{b_1,b_2,b_3=1}^{B} G_{k+1}^{(b_1,j)} G_{k'}^{(b_2,j)} G_{k''}^{(b_3,j)} P_{k+1}^{(b_1)} P_{k'}^{(b_2)} P_{k''}^{(b_3)}. \end{aligned}$$
- Case $\varepsilon =-1$ (left adjacent subband interference): As a consequence, we have for $k \ne 1$
  70 $$\begin{aligned} p_{\text{NL},k, -1 }^{(j)} (1) = \left| \gamma _3\right| ^2 \alpha ^{(1)}_{1} \sum _{k',k''=1}^{K} \sum _{b_1,b_2,b_3=1}^{B} G_{k-1}^{(b_1,j)} G_{k'}^{(b_2,j)} G_{k''}^{(b_3,j)} P_{k-1}^{(b_1)} P_{k'}^{(b_2)} P_{k''}^{(b_3)}. \end{aligned}$$
  Note that $\alpha ^{(1)}_{1}$ is involved since $\alpha ^{(1)}_{-1} = \alpha ^{(1)}_{1}$.

### Optimization problem

The second contribution of this Section is to solve the following optimization problem (with $P=1$).

#### Problem 7

71 $$\{ P_{k}^{{{ \star },(j)}} \} _{{k,j}} = \mathop {\arg \max }\limits_{{\{ P_{k}^{{(j)}} \} _{{k,j}} }} \sum\limits_{{j = 1}}^{B} {\sum\limits_{{k = 1}}^{K} {\log _{2} } } \left( {1 + Q_{k}^{{(j)}} } \right)$$

72 $$\begin{aligned}&\text {with } Q_k^{(j)} = \frac{ \mathcal{P}_{\text {L},k}^{(j)^2} + 2 \mathcal{P}_{\text {L},k}^{(j)} \mathcal{P}_{\text {LNL},k}^{(j)} + \mathcal{P}_{\text {LNL},k}^{{(j)}^2} }{ \mathcal{P}_{\text {L},k}^{(j)} \mathcal{P}_{\text {NL},k}^{(j)} + 2 \mathcal{P}_{\text {L},k}^{(j)} \mathcal{P}_{\text {INL},k}^{(j)} + \mathcal{P}_{\text {L},k}^{(j)} \mathcal{P}_{\text {I},k}^{(j)} + \mathcal{P}_{\text {L},k}^{(j)} \mathcal{P}_\text {W} - \mathcal{P}_{\text {LNL},k}^{{(j)}^2} } \end{aligned}$$

$$\text {s.t. } \, (18) \text { and } (19). $$

In the next section, we will see that the coefficients $\gamma _1$ and $\gamma _3$ are real-valued and positive for our application. The use of this assumption allows to simplify the following expressions, in order to focus on the major difficulty of solving Problem 7, where the real parts and the modulus involved in Eq. (51) have been removed since all the corresponding terms are now real-valued. Nevertheless, the proposed resolution method remains general and can be extended to other systems where these coefficients remain complex-valued.^1^

Thanks to the monotonic growth of the logarithm function, Problem 7 is equivalent to the following one.

#### Problem 8

73 $$\begin{aligned} \{P_k^{\star ,(j)}\}_{k,j}=&{\mathop {\text{ arg max}}\limits _{\{P_k^{(j)}\}_{k,j}}}\, \prod _{j=1}^B \prod _{k=1}^K \left( 1+ Q_k^{(j)}\right) \end{aligned}$$

$$\text {s.t. } \, (18) \text { and } (19). $$

First of all, we need to characterize Problem 8.

All the constraints are linear in $\textbf{P}$. In addition, one can see that all the terms $\{\alpha ^{(m)}_\ell \}_{\ell =0,1; m=1,2}$ are positive. Moreover one can easily check that $\beta$ is also positive. Consequently all the terms $\mathcal{P}_{\text {L},k}^{(j)}$, $\mathcal{P}_{\text {I},k}^{(j)}$, $\mathcal{P}_{\text {LNL},k}^{(j)}$, $\mathcal{P}_{\text {INL},k}^{(j)}$, and $\mathcal{P}_{\text {NL},k}^{(j)}$ are posynomial [18, 20, 36]. Due to the sign minus in the denominator of $Q_k^{(j)}$, Problem 8 boils down to the so-called *Signomial Programming*. Some papers in the literature [37, 38] have used the Signomial Programming to fix their optimization problem but their problem were different from ours (either linear interference or frame design).

By mimicking the approach introduced in [20], the resolution of Problem 8 leads to the following steps:

- Step 1) We put the signomial cost function into the constraints by adding auxiliary positive variables $\{t_{k}^{(j)}\}_{k,j}$ and then replacing the maximization by a minimization by taking the inverse. Then we have the equivalent Problem 9.

#### Problem 9

74 $$\begin{aligned} \{P_k^{\star ,(j)}, t_k^{\star ,(j)}\}_{k,j}=&{\mathop {\text{arg max}}\limits _{\{ P_k^{(j)},t_k^{(j)}\}_{k,j}}}\, \prod _{j=1}^B \prod _{k=1}^K t_{k}^{{(j)}^{-1}} \end{aligned}$$

$$\text {s.t. } \, (18) \text { and } (19), $$

75 $$\begin{aligned}&0 \le t_k^{(j)} \le 1+Q_k^{(j)}, \quad \forall k,j. \end{aligned}$$

- Step 2) The second idea is to transform the constraints which correspond to a ratio of signomial functions as a ratio of posynomial functions. As the denominator of $Q_k^{(j)}$ is always positive (see the first item after Eq. (51)), we replace Eq. (75) with
  76 $$\begin{aligned}{} & {} -t_k^{(j)}\le 0, \quad \forall k,j, \end{aligned}$$
  77 $$\begin{aligned}{} & {} \frac{ t_{k}^{(j)} \left( \mathcal{P}_{\text {NL},k}^{(j)} + 2 \mathcal{P}_{\text {INL},k}^{(j)} + \mathcal{P}_{\text {I},k}^{(j)} + \mathcal{P}_\text {W} \right) }{ \mathcal{P}_{\text {NL},k}^{(j)} + 2 \mathcal{P}_{\text {INL},k}^{(j)} + \mathcal{P}_{\text {I},k}^{(j)} + \mathcal{P}_\text {W} + \mathcal{P}_{\text {L},k}^{(j)} + 2 \mathcal{P}_{\text {LNL},k}^{(j)} + t_{k}^{(j)} \mathcal{P}_{\text {L},k}^{{(j)}^{-1}} \mathcal{P}_{\text {LNL},k}^{{(j)}^2}} \le 1, \quad \forall k,j. \end{aligned}$$
- Step 3) Now Problem 9 with new constraints (76) and (77) is a *Complementary Geometric Programming* since the constraints are a ratio of posynomial functions. The optimization problem will be Geometric Programming –GP– (and so can be solved optimally by resorting convex optimization toolbox [39]) if this ratio of posynomials was just a posynomial which could occur if the denominator was a monomial. In addition SCA is a suboptimal iterative approach to solve nonconvex optimization problem. Therefore we combine both techniques here. We use the SCA principle on the constraint (77): at iteration *i*, we approximate around the point $\{P_k^{(j)}(i),t_k^{(j)}(i)\}_{k,j}$ (which is the solution of the optimization problem solved at the previous iteration) the denominator of (77), denoted by $D_{k}^{(j)}(\{P_{k'}^{(j')} \}_{k',j'}, t_k^{(j)} )$, by a monomial function, denoted by $\widetilde{D}_{k,i}^{(j)}(\{P_{k'}^{(j')} \}_{k',j'}, t_k^{(j)} )$ satisfying the SCA conditions [28]. The closed-form expression for $\widetilde{D}_{k,i}^{(j)}(\{P_{k'}^{(j')}\}_{k',j'}, t_k^{(j)} )$ is reported in Appendix. So at each SCA iteration, we have a GP which is solved efficiently by convex optimization toolbox [39].

The complexity of this algorithm is not studied here, however it is reduced to the resolution of a finite sequence of GP problems. The study of the complexity and convergence of GP problems is done in [30, 31].

## Results and discussions

For the simulation scenarios, we consider $P=5$ operators, each having a multibeam satellite communication system composed of $B=2$ beams and $K=6$ users per beam. The total bandwidth is 2 GHz and each beam uses the same frequency band. The users belonging to the same beam use the FDMA technique. The size of the beam on the Earth depends on the bandwidth, the altitude (here, GSO), and the antenna size (here, a diameter of 3m) [21]. The longitudes of the ($P=5$) satellites are $9^{\circ }$, $28^{\circ }$, $42^{\circ }$, $51^{\circ }$, and $63^{\circ }$ respectively. When we focus on the single operator scenario, the satellite longitude is $28^{\circ }$. The satellite antenna radiation pattern is given by the model proposed in [40]. The FSS users are randomly drawn inside the beams, with perfect antenna pointing toward the relevant satellite. So their elevation and azimuth angles depend on their positions and that of the relevant satellite. The FSS antenna radiation pattern is given by the ITU recommendation [41]. The maximum power per user is 47dBm. The shaping filter is Square-Root Raised Cosine filter with roll-off factor 0.25. We set the operator weight to $\omega _p = 1$ for all operators. Concerning the primary system, the FS are randomly drawn given a target density. Their antenna azimuth are also randomly drawn. The FS antenna radiation pattern is given by the ITU recommendation [42]. The interference-temperature is fixed to $-90$dBm. The channel gains $\left\{ G_{k}^{(b,j,p)} \right\} _{k,b,j,p}$ and $\left\{ F_k^{(j,p,\ell )}\right\} _{k,j,p,\ell }$ depend on the antenna radiation patterns and the distances, and are evaluated as in [6]. The optimization problems are solved using CVX toolbox [39].

In addition, we add a variable gain pre-amplifier just before the HPA. This device allows to set the HPA regime by changing the channel gains (and so input powers) uniformly for incoming signal of the same antenna. For simplicity, we assume that the gains of the pre-amplifiers are identical for all HPAs and are denoted by $G_\text {amp}$.

We first consider the linear case when the nonlinear interference vanishes ($\gamma _1=1$ and $\gamma _3=0$).

**Fig. 2:**
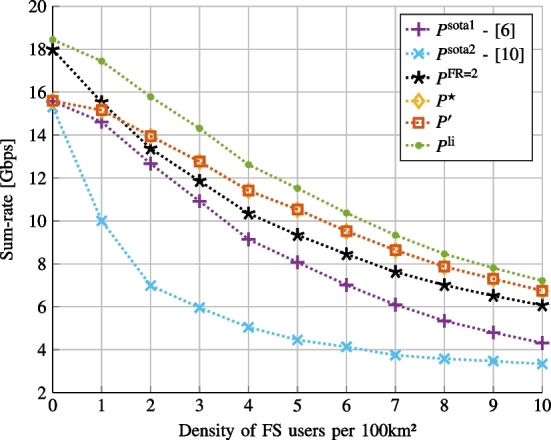
Sum-rate vs. FS density

In Fig. 2, we consider the single operator case (see Sect. 3). We actually plot the sum-rate versus FS density for various power allocations:

(i) $P^\text {sota1}$ and $P^\text {sota2}$ are the solutions provided in [6] and [10] respectively.
(ii) $P^\text {FR=2}$ is the optimal solution when we consider a frequency reuse equal to 2 (as in [43]), allowing to avoid inter-beam interference. Indeed in that case, the data rate of user *k* in beam *j* becomes $R_k^{(j)}= \frac{1}{2} \log _2\left( 1 + \frac{ 2 G_k^{(j)} P_k^{(j)} }{\mathcal{P}_W}\right)$, which is a concave function in $\textbf{P}$. We use CVX toolbox [39] to solve the resulting convex problem and find the optimal solution, denoted $P^{\text {FR=2}}$. Then the performance is evaluated using the above-mentioned data rate.
(iii) $P^{\star }$ is the solution of Problem 2 obtained with CVX toolbox [39].
(iv) $P^{\prime }$ is the solution obtained using the proposed iterative algorithm on Problem 3.
(v) $P^\text {li}$ is the SCA-based solution of Problem 1 with $P=1$ and the data rate given by Eq. (21).

The SCA algorithm outperforms the other approaches. Nevertheless, the gap decreases with the densification of the primary system justifying the use of simplified utility function. The proposed iterative approach relying on successive waterfilling offers the same performance as the optimal one when inter-beam interference is neglected. Therefore the proposed algorithm is a good trade-off between complexity and performance. By increasing the frequency reuse, the inter-beam interference is avoided and the algorithm to implement is much simpler and performs well only for low density primary network.

**Fig. 3:**
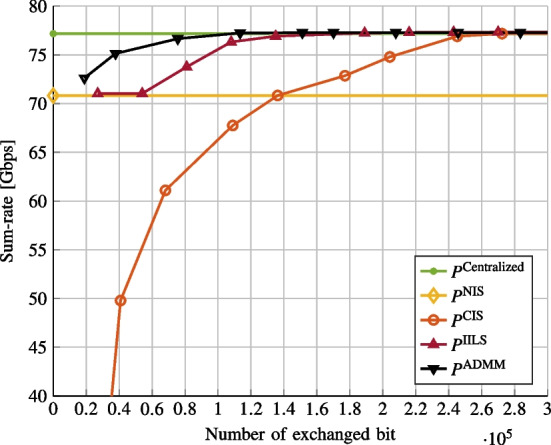
Sum-rate versus the number of exchanged bits

In Fig. 3, we consider the multiple operator case (see Sect. 4), namely, $P=5$. We consider 4 FSs per 100km$^2$. We set the maximum number of iteration for IILS and ADMM to 5. We plot the sum-rate versus the number of exchanged bits. The IILS and ADMM approaches offer good performance and are close to the centralized solution even when a small amount of exchanged bits are considered. The CIS dramatically degrades performance when the number of exchanged bits is small and so the approaches sharing interference level are more robust.

We now consider the case when the nonlinear interference does not vanish ($\gamma _1 = 1$ and $\gamma _3 = 0.05$).

**Fig. 4:**
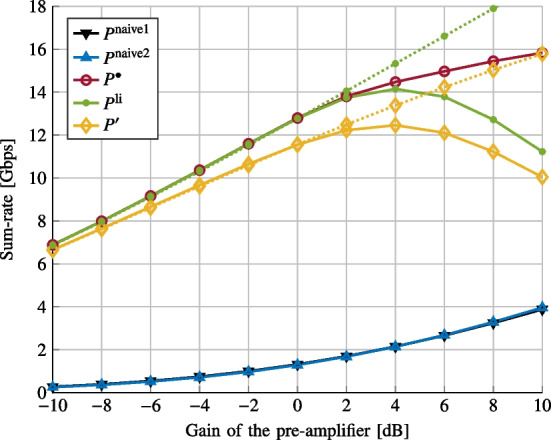
Sum-rate vs. pre-amplifier gain $G_\text {amp}$

In Fig. 4, we plot the sum-rate versus the pre-amplifier gain $G_{\text {amp}}$ for various power allocations. We consider 4 FSs per 100km$^2$. The considered power allocations are the following ones:

(i) $P^\text {naive1}$ where we force $P_k^{(j)}$ to be identical and we optimize the unique transmit power.
(ii) $P^\text {naive2}$ where we force $G_k^{(j)}P_k^{(j)}$ to be identical and we optimize the unique received power.
(iii) $P^{\bullet }$ The SCA-based solution of Problem 9.
(iv) $P^\text {li}$ the SCA-based solution of Problem 1 with $P=1$ and the data rate given by Eq. (21) where the nonlinearity is not taken into account.
(v) $P^{\prime }$ the solution obtained using the proposed iterative algorithm on Problem 3, which does not take into account the nonlinearity.

The solid lines correspond to the evaluation of the sum-rate given by Eq. (51), while the dashed lines correspond to the evaluation of the sum-rate given by Eq. (21). We observe that the proposed solution $P^{\bullet }$ enables us to increase the sum-rate when the nonlinear effect can not be neglected, and outperforms the naive solutions.

**Fig. 5:**
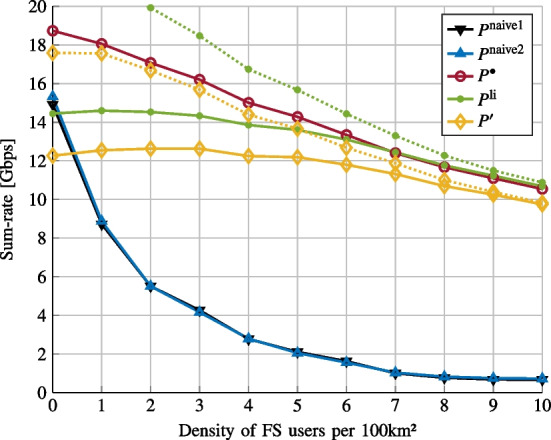
Sum-rate vs. FS density

In Fig. 5, we plot the sum-rate versus FS density for the power allocations described on Fig. 4. We consider $G_{\text {amp}}=6$dB. Once again, the proposed approach provides gain in performance. This gain nevertheless decreases when the FS density increases since in that case, the interference temperature constraints lead to decrease the transmit powers and so the induced nonlinear effect on the satellite.

## Concluding remarks

We have developed algorithms for power allocation in the context of multi-operator multibeam uplink satellite communications seen as secondary system when a terrestrial primary system operates. The main contributions of the paper are twofold: we provided a distributed allocation when the operators have to coordinate their own power allocation; we provided new power allocation when nonlinear devices are taken into account.

## Data Availability

Data sharing is not applicable to this article as no datasets were generated or analysed during the current study

## Appendix

### Approximation of $D_{k}^{(j)}$ at point $\left( \{P_{k'}^{(j')}(i)\}_{k',j'}, t_k^{(j)}(i) \right)$

Like [36], we use the trick that the arithmetic mean can be lower-bounded by the geometric mean. As a consequence, we have

78

with $$D_{k}^{(j)}(i):= D_k^{(j)} \left( \{P_{k^{\prime}}^{(j^{\prime})}(i)\}_{k^{\prime},j^{\prime}}, t_k^{(j)}(i) \right)$$ and

79

## Abbreviations

ADMM: Alternate direction multiplier method
AWGN: Additive white gaussian noise
CGP: Complementary geometric programming
CIS: Channel information sharing
CR: Cognitive radio
FS: Fixed service
FSS: Fixed satellite service
GP: Geometric programming
GSO: Geostationary
HPA: High power amplifier
IILS: Iterative interference level sharing
MF–TDMA: Multiple-frequency time-division multiple access
NIS: No information sharing
SatCom: Satellite communication
SCA: Successive convex approximation
SP: Signomial programming
SRRC: Square-root raised cosine

## Footnotes

1 Indeed, one can easily extend the resolution method for complex-valued coefficients, where the sign of $\Re \{\gamma _1 \overline{\gamma _3}\}$ must be investigated.
